# Occurrence and bacterial loads of *Bartonella* and haemotropic *Mycoplasma* species in privately owned cats and dogs and their fleas from East and Southeast Asia

**DOI:** 10.1111/zph.12959

**Published:** 2022-05-11

**Authors:** Aya Attia Koraney Zarea, Marcos Antonio Bezerra‐Santos, Viet‐Linh Nguyen, Vito Colella, Filipe Dantas‐Torres, Lenaig Halos, Frederic Beugnet, Maria Tempesta, Domenico Otranto, Grazia Greco

**Affiliations:** ^1^ Department of Veterinary Medicine University of Bari “Aldo Moro” Bari Italy; ^2^ Department of Microbiology and Immunology, Veterinary Research Institute National Research Centre (NRC) Cairo Egypt; ^3^ Oxford University Clinical Research Unit (OUCRU) Ho Chi Minh City Vietnam; ^4^ Faculty of Veterinary and Agricultural Sciences University of Melbourne Melbourne Victoria Australia; ^5^ Department of Immunology Aggeu Magalhães Institute Recife Brazil; ^6^ Boehringer Ingelheim Animal Health Lyon France; ^7^ Bill & Melinda Gates Foundation Seattle Washington USA; ^8^ Faculty of Veterinary Sciences Bu‐Ali Sina University Hamedan Iran

**Keywords:** *Bartonella* spp*.*, *Candidatus* Bartonella merieuxii, flea, East and Southeast Asia, haemoplasmas

## Abstract

*Bartonella* spp. and haemoplasmas are pathogens of veterinary and medical interest with ectoparasites mainly involved in their transmission. This study aimed at molecular detection of *Bartonella* spp. and haemoplasmas in cats (*n* = 93) and dogs (*n* = 96), and their related fleas (*n* = 189) from countries in East and Southeast Asia. *Ctenocephalides felis* was the dominant flea species infesting both cats (97.85%) and dogs (75%) followed by *Ctenocephalides orientis* in dogs (18.75%) and rarely in cats (5.2%). *Bartonella* spp. DNA was only detected in blood samples of flea‐infested cats (21.51%) (*p* < .0001, OR = 27.70) with *Bartonella henselae* more frequently detected than *Bartonella clarridgeiae* in cat hosts (15.05%, 6.45%) and their associated fleas (17.24%, 13.79%). Out of three *Bartonella*‐positive fleas from dogs, two *Ct. orientis* fleas carried *Bartonella vinsonii* subsp. *berkhoffii* and *Bartonella clarridgeiae*, while the 3rd flea (*Ct. felis*) carried *Candidatus* Bartonella merieuxii*.* Felines represented a risk factor for *Bartonella* spp. infections, where fleas collected from cats (32.25%) presented an increased likelihood for *Bartonella* spp. occurrence (*p* < .0001, OR = 14.76) than those from dogs (3.13%). Moreover, when analysing infectious status, higher *Bartonella* spp*.* DNA loads were detected in fleas from bacteraemic cats compared to those from non‐bacteraemic ones (*p* < .05). The haemoplasma occurrence was 16.13% (15/93) and 4.17% (4/96) in cat and dog blood samples from different countries (i.e. Indonesia, Malaysia, the Philippines, Taiwan and Thailand), with cats more at risk of infection (*p* < .01, OR = 5.96) than dogs. Unlike *Bartonella* spp., there was no evidence for flea involvement in the hemoplasmas' transmission cycle, thus supporting the hypothesis of non‐vectorial transmission for these pathogens. In conclusion, client‐owned cats and dogs living in East and Southeast Asia countries are exposed to vector‐borne pathogens with fleas from cats playing a key role in *Bartonella* spp. transmission, thus posing a high risk of infection for humans sharing the same environment.

**Impacts**

- *Bartonella* and haemoplasmas are pathogens impacting animal and public health.
- Companion animals living in East and Southeast Asia countries are exposed to *Bartonella* and haemoplasma infections with cats more at risk than dogs.
- Fleas serve as active vectors of *Bartonella* spp., but unlikely of haemoplasmas.

## 1 INTRODUCTION

Asia is experiencing a rapid increase in the number of dogs and cats kept as family pets. Although these animals provide substantial positive psychological and physiological benefits to their owners (Chongsuvivatwong et al., 2011), companion dogs and cats might act as reservoirs of several zoonotic agents and represent a risk to human health in Asia (Barrs et al., 2010; Colella et al., 2020; Duong et al., 2016; Kosoy & Goodrich, 2019; Nguyen et al., 2020). Moreover, climatic and environmental conditions of East (EA) and Southeast Asia (SEA) countries are suitable for the arthropod proliferation, including fleas that are often involved in the transmission of pathogens of medical and veterinary interest (Chandra et al., 2017; Chomel et al., 1996; Watanabe, 2012; Yuan et al., 2011). Recently, a large proportion of companion cats (19.6%) and dogs (14.8%) were diagnosed with flea infestation in a large survey conducted in EA and SEA (Colella et al., 2020). The fleas' role of dogs and cats in transmitting several bacterial, viral or parasitic pathogenic agents has been widely recognized (Bezerra‐Santos et al., 2021; Chandra et al., 2017; Chomel et al., 1996; Rolain, Franc, et al., 2003).

Among the vector borne bacteria, the genus *Bartonella* includes different species of concern for the health of animals and humans (Breitschwerdt et al., 2010; Chomel et al., 2006). In particular, domestic cats act as mammal reservoirs for *B. henselae*, and other species including *B. clarridgeiae* and *B. koehlerae* (Breitschwerdt & Kordick, 2000; Chomel et al., 1996, 2006; Rolain, Fournier, et al., 2003). After the infection, cats develop a long lasting (from weeks to months) mainly asymptomatic intraerythrocytic bacteraemia (Guptill et al., 1997), acting as a source of infection for the fleas including *Ctenocephalides felis*, that is the active vector for different *Bartonella* spp. including *B. henselae*, *B. clarridgeiae* and putatively *B. koehlerae* (Chomel et al., 1996; Greco, Brianti, et al., 2019; Rolain, Franc, et al., 2003). Transmission pathways of *Bartonella* spp. to humans include contamination of wounds with flea dropping. These infections, commonly named “cat scratch disease”, may cause mild self‐limiting to life‐threatening syndromes such as fever, fatigue, lymphadenopathy and less commonly endocarditis, meningitis or encephalitis (Breitschwerdt & Kordick, 2000; Pitassi et al., 2015; Vieira‐Damian et al., 2015).

Dogs can also harbour several *Bartonella* species including *B. henselae*, *B. vinsonii* subsp*. berkhoffii*, *C*. B. merieuxii and *B. rochalimae* (Breitschwerdt et al., 2010; Chomel et al., 2012; Chomel, Boulouis, et al., 2009; Diniz et al., 2007; Greco, Sazmand, et al., 2019). Like humans, infected dogs may also develop severe disease manifestations including endocarditis, splenomegaly or vasculitis (Álvarez‐Fernández et al., 2018; Chomel et al., 2006; Chomel, Kasten, et al., 2009).

Several studies detected the occurrence of *Bartonella* spp. in cats, dogs and fleas from EA and SEA with prevalence of up to 60 % (Assarasakorn et al., 2012; Chang et al., 2006; Chomel et al., 1999; Inoue et al., 2009; Jensen et al., 2000; Kim et al., 2009; Maruyama et al., 2001; Singer et al., 2020; Yuan et al., 2011; Zhang et al., 2019). However, no studies have investigated the relative contribution of dogs, cats and fleas in the transmission cycle of *Bartonella* spp. in the area.

Haemotropic mycoplasmas (“haemoplasmas”) are not‐yet cultured bacteria of the genus *Mycoplasma* (Neimark et al., 2001). Based on phylogeny (rather than pathogenicity or host specificity), haemoplasmas are split into two groups, namely the haemominutum group and the haemofelis group (Peters et al., 2008; Tasker, Helps, Day, Harbour, et al., 2003). *Mycoplasma haemofelis* (*Mhf*), *Candidatus* Mycoplasma haemominutum (*C*Mhm) and *Candidatus* Mycoplasma turicensis (*C*Mt) are the species mainly detected in cats, while *Mycoplasma haemocanis* (*Mhc*) and *Candidatus* M. haematoparvum (*C*Mhp) are detected in dogs (Messick et al., 2002; Sykes et al., 2004; Sykes et al., 2005; Willi et al., 2006). These microorganisms attach and grow on the surfaces of the erythrocytes causing from chronic infections to life‐threatening haemolytic anaemia (Kirchhoff et al., 1984; Messick, 2004; Sykes, 2010; Tasker, 2010). Furthermore, the detection of *Mycoplasma haemofelis*‐like organisms and *C*Mhp in HIV‐positive immunocompromised patients from Brazil and Africa raises questions on the zoonotic potential of these pathogens (Dos Santos et al., 2008; Maggi et al., 2013; Tasker et al., 2010).

Currently, the transmission route of haemoplasmas remains a matter of debate, although fleas or ticks have been hypothesized as natural vectors (Novacco et al., 2010; Senevtratna et al., 1973; Woods et al., 2005). Nevertheless, direct transmission through bites and blood transfusion have also been described (Tasker, 2010; Willi et al., 2007). Few studies have investigated the occurrence of haemoplasmas in EA and SEA, and have reported high prevalence in community dogs (40%), stray cats (23% to 38%) and client‐owned cats (23%) and their fleas (34%) from Thailand (Assarasakorn et al., 2012; Do et al., 2020; Huggins et al., 2019; Kaewmongkol et al., 2017) as well as in free‐ranging dogs (~13%) from Cambodia (Huggins et al., 2021; Inpankaew et al., 2016).

This study aimed to investigate the occurrence of *Bartonella* spp. and haemoplasmas in dogs, cats and fleas to understand their relative contribution in the epidemiology of these pathogens in East and Southeast Asia.

## 2 MATERIALS AND METHODS

### 2.1 Study area and samples

Animals and fleas included in the study represent a randomly selected sub‐sample (*n* = 189; 92 cats and 93 dogs) from a larger number of animals enrolled in a previous multi‐centre survey consisting of privately owned animals (i.e. 1229 dogs and 1152 cats) from China, Indonesia, Malaysia, the Philippines, Singapore, Taiwan, Thailand and Vietnam (Colella et al., 2020; Nguyen et al., 2020). Animal selection was performed according to the presence of flea infestation. The minimum sample size (92 cats and 93 dogs) was estimated based on the assumptions of the confidence level of 95%, an accepted error of 7% and a minimum expected prevalence of 15% for *Bartonella* species/haemotropic *Mycoplasma* infections. Blood samples and fleas (one for each animal) were collected from the animals (*n* = 93 cats, 96 dogs). Each animal was infested with a range of 1 to 3 fleas. Animals with history of regular outdoor access and having not received recent antiparasitic treatments were enrolled. Data on the animal age, gender, clinical signs and flea species were recorded. All fleas were molecularly and morphologically identified at the species level as described elsewhere (Colella et al., 2020).

### 2.2 Molecular investigation for *Bartonella* spp. and haemoplasmas

#### 2.2.1 DNA extraction

Blood (100 μl) and flea (individual) samples were subjected to the extraction of genomic DNA using protocols previously described (Colella et al., 2020; Nguyen et al., 2020). DNA was eluted in 100 μl of AE buffer and carefully quantified using the fluorometric Qubit® dsDNA HS (High Sensitivity) Assay kit, DNA (10 μl) from each sample was used to run the qPCR/cPCR assays listed in Table 1. Animal species DNA targets were amplified using dog's SSR and cat's SSR primers respectively (Abdel‐Rahman et al., 2009).

**TABLE 1:** Target and primers used in this study

Target	Target gene	Primer name	bp	%Reaction efficiency (R^2^)	References
*Bartonella* genus	ssrA	ssrA‐F: GCTATGGTAATAAATGGACAATGAAATAA	300	94.84 (0.99)	Diaz et al., 2012
		ssrA‐R: GCTTCTGTTGCCAGGTG			
		Probe: ACCCCGCTTAAACCTGCGACG			
	ITS	325‐F: CTTCAGATGATGATCCCAAGCCTTYTGGCG	408–673		Diniz et al., 2007
		1100‐R: GAACCGACGACCCCCTGCTTGCAAAGC A			
*B. henselae*	pap31	Bh‐F: TAAGGTTGAAATAACTGATCCGAA T			Diniz et al., 2007
		668‐R: CACCACCAGCAAAATAAGGCATMAY			
*B. henselae* typing	16S	16S‐F: AGAGTTTGATCCTGGCTCAG	185		Bergmans et al., 1996; Sander et al., 1998
		BH1‐R: CCGATAAATCTTTCTCCCTAA			
		BH2‐R: CCGATAAATCTTTCTCCAAAT			
Haemoplasmas spp.	16S rRNA	HBT‐F: ATACGGCCCATATTCCTACG	595–618		Criado‐Fornelio et al., 2003
		HBT‐R: TGCTCCACCACTTGTTCA			
Haemofelis group	16S rRNA	F: GGAGCGGTGGAATGTGTAG	114	98.2 (0.99)	Tasker et al., 2010
		R: GGGGTATCTAATCCCATTTGC			
		Probe: TYAAGAACACCAGAGGCGAAGGCG			
Haemominutum group	16S rRNA	F: GGGGCCAAGTCAAGTCATC	139	97.4 (0.99)	
		R: GCGAATTGCAGCCTTTTATC			
		Probe: TACCATTGTAGCACGTTYGCAGCCC			
Cat	SSR	F: CTCATTCATCGATCTACCCA	672		Abdel‐Rahman et al., 2009
		R: GTGAGTGTTAAAACTAGTACTAGAAGA			
Dog	SSR	F: GGAGTATGCTTGATTCTACAG	808		
		R: AGAAGTGGAATGAATGCC			

#### 2.2.2 Molecular detection, quantification and identification of *Bartonella* spp

All DNA samples were subjected to the molecular screening using *Bartonella* genus‐specific quantitative real‐time PCR (qPCR) assay targeting the transfer‐mRNA *ssr*A (*ssr*A) gene (Diaz et al., 2012) (Table 1). Furthermore, *Bartonella* DNAs loads for each flea and blood sample were calculated by using the standard curve generated with different 10‐fold dilutions (0.1 Log_10_ to 9 Log_10_ copies per 10 μl) of the plasmid DNA encoding a 300‐bp *B. henselae ssr*A gene fragment. qPCR amplification was conducted in multiplate PCR plates (Bio‐Rad™) using a CFX96 Touch Real‐Time PCR Detection System (Bio‐Rad™). For *Bartonella* species identification and typing, the *ssr*A qPCR positive samples were further subjected to different additional conventional PCR (cPCR) assays, that amplify *ssr*A, 16S rRNA and 16‐23S intergenic spacer (ITS) target fragments (Table 1) (Bergmans et al., 1996; Diaz et al., 2012; Diniz et al., 2007; Sander et al., 1998). Reference strains *B. clarridgeiae* (MH348146), *B. henselae* (MH350809), *B. rochalimae* (MK780191) and *B. vinsonii* subsp. *berkhoffii* (MK773857) were used as positive controls for each cPCR. *ssr*A and ITS cPCR‐positive products were subjected to purification using the NEB Exo‐SAP PCR purification kit (New England Biolabs, Inc.) prior to the sequencing by Eurofins Genomics.

#### 2.2.3 Molecular detection, quantification and identification of the haemoplasma species

For the haemoplasmas' detection, all the DNA samples were screened by using two generic haemoplasma haemofelis and haemominutum group‐specific qPCR assays targeting the 16S rRNA (Tasker et al., 2010) (Table 1). Furthermore, haemoplasma DNA loads for each flea and blood sample were calculated by using the standard curves generated with different 10‐fold dilutions (0.1 Log_10_ to 9 Log_10_ copies per 10 μl) of the plasmid DNAs encoding the 16S rRNA fragments from *M. haemofelis* and *C*. M. haemominutum, according to protocol previously described (Tasker, Helps, Day, Gruffydd‐Jones, & Harbour, 2003). Furthermore, for haemoplasma species differentiation each positive sample was submitted to an additional 16S rRNA amplification (cPCR) (Criado‐Fornelio et al., 2003) and the products of expected sizes were purified using the NEB Exo‐SAP PCR purification kit (New England Biolabs, Inc.) and sequenced by Eurofins Genomics.

All *Bartonella* spp. and haemoplasma DNA sequences were first edited and then subjected to a preliminary analysis using Local Basic Alignment Tool (BLAST) and aligned against the closely related sequence homologous using the ClustalW application within the Geneious® 10.3.1 software package (Biomatters Ltd.).

### 2.3 Statistical analysis

An animal or flea sample was considered *Bartonella* spp. and/or haemoplasma infected if it was positive in the *ssr*A qPCR and/or in the 16S rRNA qPCR assays respectively (Diaz et al., 2012; Tasker et al., 2010). Exact binomial 95% confidence intervals (CIs) were used to calculate the infection rates. Fisher's exact / Chi squared tests with Yate's correction (χ^2^) and odds ratio (OR) were used to analyse the differences of pathogen detections in blood and flea samples and risk factors. The non‐parametric Mann–Whitney U and/or the Kruskal–Wallis tests were used to compare bacterial loads (expressed in log_10_ DNA copies/10 μl) for both *Bartonella* and haemoplasma species from animal and flea samples as well as to assess the relationships between animal host *status* and their associated fleas for each bacterial species. Significant differences were set at *p* ≤ .05. All statistical analyses were performed using ibm spss Statistics software, version 25.

## 3 RESULTS

### 3.1 Animals and ectoparasites

Out of the 93 cats (35 females and 58 males), with age ranging from 2 months to 18 years old (median: 12 months; mode: 24 months), the majority (70.97%) was from urban areas in good health status (Table 2). Out of the 96 dogs, equally distributed for gender and with age ranging from 2 months to 18 years old (median and mode: 36 months), the majority (94.79%) was in good condition (Table 2). The collected fleas were identified as *Ct. felis* in cats (91/93, 97.85%) and dogs (72/96, 75%), followed by *Ct. orientis* in dogs (23/96, 23.96%). Furthermore, *Xenopsylla* (*X*.) *cheopis* and *Ct. canis* were sporadically detected in cats (2/93, 2.14%) and dogs (1/96) (Table 2).

**TABLE 2:** Number and characteristics of animals and their fleas from East and Southeast Asia

	Cats *n* (%) *N* = 93	Dogs *n* (%) *N* = 96
Age in months (median/mode) (range)	(12/24) (2–216)	(36/36) (2–216)
Gender		
Male	58 (62.36)	46 (47.91)
Female	35 (37.63)	50 (52.17)
Husbandry		
Urban area	66 (70.97)	56 (58.33)
Rural area	27 (29.03)	40 (41.67)
Temperature		
Fever	13 (13.99)	5 (5.2)
No fever	80 (86.02)	91 (94.79)
Lymph node		
Enlarged	8 (8.06)	10 (10.42)
Normal	85 (31.39)	86 (89.67)
Flea species		
*Ct. felis*	91 (97.85)	72 (75)
*Ct. orientis*	1 (1.07)	23 (23.96)
*Ct. canis*		1 (1.04)
*Xenopsylla cheopis*	1 (1.07)	

### 3.2 Detection and quantification of *Bartonella* spp. DNA

All DNA samples were positive for the species‐specific DNA fragment, confirming the quality of DNA extraction and the absence of DNA inhibitors. The reaction efficiency of the qPCR *ssr*A assay was 94.84%, slope −3.45, *r* ^2^ .997, *y*‐intercept 41.50.

*Bartonella* spp. DNA was detected in 20/93 (21.51%, 95% CI = 13.15–29.86) cat blood samples, but in none of those of dogs (*p* < .0001, OR = 27.70) (Tables 3, 4, 5). Out of 189 fleas, 30/93 (32.26%, 95% CI = 22.76–41.76) from cats and 3/96 (3.12%, 95% CI = 0.00–6.61) from dogs were *Bartonella* spp. positive, with evidence for *Bartonella* spp. infection more frequent in fleas from cats than from dogs (*p* < .0001, OR = 14.76) (Tables 3, 4, 5). The combined *ssrA* and 16S rRNA/ITS typing revealed that *B. henselae* I was the dominant species in cat blood samples, with prevalence of 13.98% (13/93, 95% CI = 6.93–21.03) followed by *B. clarridgeiae* 6.45% (6/93, 95% CI = 1.46–11.44) and *B. henselae* II 1.08% (1/93) (Tables 3 and 4). A similar trend was observed in fleas collected from cats with *B. henselae* I (16/93, 17.20%, 95% CI = 9.53–24.88) most frequent than *B. clarridgeiae* (13/93, 13.98%, 95% CI = 6.93–21.03) and *B. henselae* II (1/93, 1.08%, 95% CI = 0.00–3.17) (Table 4). Furthermore, out of 20 bacteraemic cats, 11 were infested with *Bartonella*‐positive fleas of which 8 and 3 pairs hosting *B. henselae* I and *B. clarridgeiae*, respectively, but one cat hosting different *Bartonella* spp. than its flea (*B. henselae* I vs. *B. clarridgeiae*) (Tables 3 and 4).

**TABLE 3:** *Bartonella* and haemotropic *Mycoplasma* species detected in cats, dogs and their fleas from countries in East and Southeast Asia

No	Country	Animal and flea ID	*Bartonella* spp		*Locus*	Accession number	Haemoplasma	
			Animal host	Flea			Animal host	Flea
1	Indonesia	Cat‐25	Bh I	Bh I	ITS	ns		
2	Indonesia	Cat‐26	Bh I	Bh I	ITS	ns		
3	Indonesia	Cat‐27	Bh I	Bh I	ITS	ns		
4	Indonesia	Cat‐28	Bh I	Bh I	ITS	ns		
5	Indonesia	Cat‐31	Bh I	Bh I	ITS	ns		
6	Indonesia	Cat‐32	Bh I	Bh I	ITS	ns		
7	Indonesia	Cat‐37	*Bc*	Bc	*ssr*A	MZ327707^‡^		
8	Indonesia	Cat‐39	Bc	Bc	ITS	MZ323351^§^		
9	Indonesia	Cat‐48	Bh I	Bh I	ITS	MZ323358^‡^		
10	Indonesia	Cat‐65	Bh I	Bh I	ITS	ns		
11	Taiwan	Cat‐01	Bh I	Bc		ns		
12	Taiwan	Cat‐03	Bc	Bc	*ssr*A	MZ327706^‡^		
13	Taiwan	Cat‐44	Bh I		ITS	MZ323357^§^		
14	Philippines	Cat‐51	Bc		ITS	MZ323355^§^	*Mhf* & *C*Mhm	
15	Philippines	Cat‐103	Bc		ITS	MZ323352^§^	*Mhf*	
16	Philippines	Cat‐104	Bc			ns		
17	Malaysia	Cat‐01	Bh I			ns		
18	Malaysia	Cat‐04	Bh I			ns	*Mhf*	
19	Malaysia	Cat‐40	Bh I		ITS			
20	China	Cat‐139	Bh II			ns		
21	China	Cat‐152		Bc	ITS	MZ323354 ^‡^		
22	China	Cat‐239		Bh II		ns		
23	Singapore	Cat‐18		Bc	*ssr*A	MZ327703 ^‡^		
24	Vietnam	Cat‐30		Bh I		ns		*C*Mhm
25	Vietnam	Cat‐34		Bh I		ns		*C*Mhm
26	Vietnam	Cat‐47		Bh I		ns		
27	Taiwan	Cat‐02		Bh I		ns		
28	Taiwan	Cat‐06		Bc	ITS	MZ323353 ^‡^		
29	Philippines	Cat‐46		Bc		ns		
30	Philippines	Cat‐101		Bc		ns		
31	Indonesia	Cat‐24		Bc	ssrA			
32	Indonesia	Cat‐29		Bh I	ssrA	MZ327701 ^‡^		
33	Indonesia	Cat‐35		Bh I		ns		
34	Indonesia	Cat‐42		BhI		ns		
35	Indonesia	Cat‐53		Bc	ssrA	MZ327705 ^‡^		CMhm
36	Thailand	Cat‐78		Bh I		ns		
37	Thailand	Cat‐89		Bc	ITS	ns		
38	Philippines	Cat‐91		Bc	ITS	ns	*Mhf*	
39	Indonesia	Dog‐50		*C*Bm	*ssr*A	MZ327699 ^‡^		
40	Thailand	Dog‐96		Bvb	*ssr*A	MZ327700 ^‡^		
41	Thailand	Dog‐105		Bc	*ssr*A	MZ327704 ^‡^	Mhc	
42	Malaysia	Cat‐14					*C*Mhm	
43	Indonesia	Cat‐13					*C*Mhm	
44	Indonesia	Cat‐36					*C*Mhm	
45	Philippines	Cat‐07					*C*Mhm	
46	Philippines	Cat‐40					Mhf	
47	Philippines	Cat‐41					*C*Mhm	
48	Philippines	Cat‐42					Mhf	
49	Philippines	Cat‐58					Mhf	
50	Philippines	Cat‐105					*C*Mhm	
51	Taiwan	Cat‐14					*C*Mhm	
52	Taiwan	Cat‐41					*C*Mhm	
53	Indonesia	Cat‐44						*C*Mhm
54	Vietnam	Cat‐09						*C*Mhm
55	Vietnam	Cat‐29						*C*Mhm
56	Vietnam	Cat‐38						*C*Mhm
57	Taiwan	Dog‐45					*Mhc*	
58	Taiwan	Dog‐93					*Mhc*	
59	Philippines	Dog‐02					*Mhc*	
60	Philippines	Dog‐40						*C*Mhp

*Note*: Bh I, *B. henselae* genotype I; Bh II, *B. henselae* genotype II; Bc: *B. clarridgeiae*; *B*vb: *B. vinsonii* subsp. *berkhoffii*; *C*Bm: *Candidatus* Bartonella merieuxii *C*Mhm: C*andidatus* Mycoplasma haemominutum*; C*Mhp: C*andidatus* Mycoplasma haematoparvum; *Mhc*: *Mycoplasma canis*; *Mhf*: *Mycoplasma haemofelis. Locus* submitted for sequence analyses from flea (‡), and animal host (§).

Abbreviation: ns, not submitted.

**TABLE 4:** Occurrence of Bartonella spp. and haemotropic Mycoplasma spp. from cats, dogs and their fleas from East and Southeast Asia

Pathogen	Prevalence							
	Cats *n* (%)				Dogs *n* (%)			
	Blood samples (*n* = 93)	CI (95%)	Flea (*n* = 93)	CI (95%)	Blood samples (*n* = 96)	CI (95%)	Flea (*n* = 96)	CI (95%)
Prevalence for any pathogen	32 (34.41)	24.75–44.06	35 (37.63)	27.79–47.48	4 (4.17)	0.17–8.16	4 (4.17)	0.17–8.16
*Bartonella* spp.	20 (21.51)	13.15–29.86	30 (32.26)	22.76–41.76			3 (3.13)	0.00–6.61
*B. henselae*	14 (15.05)		17 (18.28)					
*B. henselae* I	13 (13.98)	6.93–21.03	16 (17.20)		9.53–24.88			
*B. henselae* II	1 (1.08)	0.00–3.17	1 (1.08)	0.00–3.17				
*B. clarridgeiae*	6 (6.45)	1.46–11.44	13 (13.98)		6.93–21.03		1 (1.04)	0.00–3.07
*B. vinsonii* subsp*. berkhoffii*							1 (1.04)	0.00–3.07
*C*.B. merieuxii							1 (1.04)	0.00–3.07
Haemotropic *Mycoplasma* spp.	15 (16.13)	8.65–23.60	7 (7.53)	2.16–12.89	4 (4.17)	0.17–8.16	1 (1.04)	0.00–3.07
*C*Mhm	8 (8.6)	2.90–14.30	7 (7.53)					
*Mhf*	6 (6.45)	1.46–11.44						
*C*Mhm & *Mhf*	1 (1.08)	0.00–3.17						
*Mhc*					4 (4.17)	0.17–8.16		
*C*Mhp							1 (1.04)	0.00–3.07
Mixed infections								
*B. henselae* I & *C*Mhm			2 (2.15)	0.00–5.10				
*B. henselae* I & *Mhf*	1 (1.08)	0.00–3.17						
*B. clarridgeiae* & *Mhf*	1 (1.08)	0.00–3.17						
*B. clarridgeiae*, *Mhf* & *C*Mhm	1 (1.08)	0.00–3.17						

**TABLE 5:** Association between pathogen status and variables

Variable	Category	Frequency (*n*)	Prevalence (%) CI	Fischer's *p* Value	χ2	OR	CI 95% OR
Animal species harbouring at least one m.o	Cat	32/93	34.41 (24.75–44.06)	**.0001**		**12.056**	**4.06–35.84**
	Dog	4/96	4.17 (0.17–8.16)	Ref			
Fleas harbouring at least one m.o	Cat fleas	35/93	37.63 (27.79–47.48)	**.0001**		**13.897**	**4.69–41.09**
	Dog fleas	4/96	4.17 (0.17–8.16)	Ref			
*Bartonella* spp.							
Animal source	Cat	20/93	21.51 (13.15–29.86)	**.0001**		**27.70**	**3.64–210.20**
	Dog	0/96	—	Ref			
*Bartonella* spp. in cat							
Age	≤1	8/46	17.39 (6.44–28.34)	Ref			
	≥1	12/47	25.5 (13.7–38)	.33			
Husbandry	Urban	12/66	18.18 (21.96–44.71)	.22			
	Rural	8/27	29.6 (12.41–46.85)	Ref			
Gender	Male	12/58	20.69 (10.26–31.11)	.8			
	Female	8/35	22.86 (8.95–36.77)	Ref			
Fever	yes	2/13	15.38 (0.00–35.00)	Ref			
	no	18/80	22.5 (13.35–31.65)	.5			
Lymph node	Enlarged	4/8	50 (15.35–84.65)	.13			
	Normal	16/85	18.82 (10.51–27.13)	Ref			
Animal source of positive fleas	Cat	30/93	32.26 (22.76–41.76)	**.0001**		**14.76**	**4.31–50.46**
	Dog	3/96	3.125 (0.00–6.61)	Ref			
Bacteraemic cats harbouring infected fleas	Yes	12/20	60 (38.53–81.47)	**<.0001**	**22.37**	**12.19**	**3.83–38.78**
	No	8/73	10.96 (3.79–18.12)	Ref			
Haemoplasmas spp.							
Animal source	Cat	15/93	16.13% (8.65–23.60)	**.006**		**5.96**	**1.66–21.35**
	Dog	4/96	3.13 (0.17–8.16)	Ref			
Haemoplasmas in cats							
Age	≤1	5/46	10.87 (1.87–19.86)	Ref			
	≥1	10/47	21.28 (9.58–32.98)	.172			
Husbandry	Urban	10/66	15.15 (6.50–23.80)	.68			
	Rural	5/27	18.52 (3.87–33.17)	Ref			
Gender	Male	10/58	17.24 (7.52–26.96)	.70			
	Female	5/35	14.29 (2.69–25.88)	Ref			
Fever	Yes	0/13		Ref			
	No	15/80	18.75 (10.20–27.30)	.088			
Animal source of positive fleas	Cat	7/93	7.53 (2.16–12.89)	**.027**		**7.73**	**0.93–64.13**
	Dog	1/96	1.04 (0.00–3.07)				
Bacteraemic cats harbouring infected fleas	Yes	0/15	—	—			
	No	0/78					
Bacteraemic dogs harbouring infected fleas	Yes	0/4	—	—			
	No	1/92	1.09 (0.00–3.21)				

*Note:* Significant values are displayed in bold.

Abbreviation: m.o: microorganism.

Out of the 3 *Bartonella* spp.‐positive fleas from dogs, two from Thailand, *Ct. orientis* (#40) and *Ct. felis* (#41), carried *B. vinsonii* subsp. *berkhoffii* III and *B. clarridgeiae* DNA respectively (Tables 3 and 4). The positive flea (*Ct. felis*) (#39) from Indonesia carried *C*. B. merieuxii DNA displaying the *ssr*A sequence identical to clones previously detected from domestic and wild canids in Iran, Iraq and Italy (Chomel et al., 2012; Greco et al., 2021; Greco, Sazmand, et al., 2019) (Table 3).

The *Bartonella* spp. bacterial loads determined in the positive cats ranged from 1.03 to 4.28 Log_10_ (mean: 2.33 ± SD 0.88; median: 2.28) DNA copies/10 μl with no significant differences for gender (Mann–Whitney *U* test [MWt], *p* = .69], age (MW, *p* = .91), and the presence for sign of fever (MWt, *p* = .674) or enlarged lymph nodes (MWt, *p* = .12). Furthermore, no differences in bacterial loads were observed between the cats according to the identified *Bartonella* species (MWt, *p* = .84).

The bacterial loads in *Bartonella*‐infected fleas from cats ranged from 1.18 to 7.33 log_10_ (mean: 3.62 ± SD 1.97; median: 3.29) DNA copies/10 μl, with no statistically significant association with gender (MWt, *p* = .13). Furthermore, although there was no statistically significant association (*p* = .065), *B. henselae*‐infected fleas displayed higher bacterial loads (0.65 to 7.33 log_10_ [mean: 4.08 ± SD 2.05; median: 4.9] DNA copies/10 μl) than those infected with *B. clarridgeiae* (0.68 to 7.21 log_10_ [mean: 2.72 ± SD 1.97; median: 2.02] DNA copies/10 μl).

**FIGURE 1:**
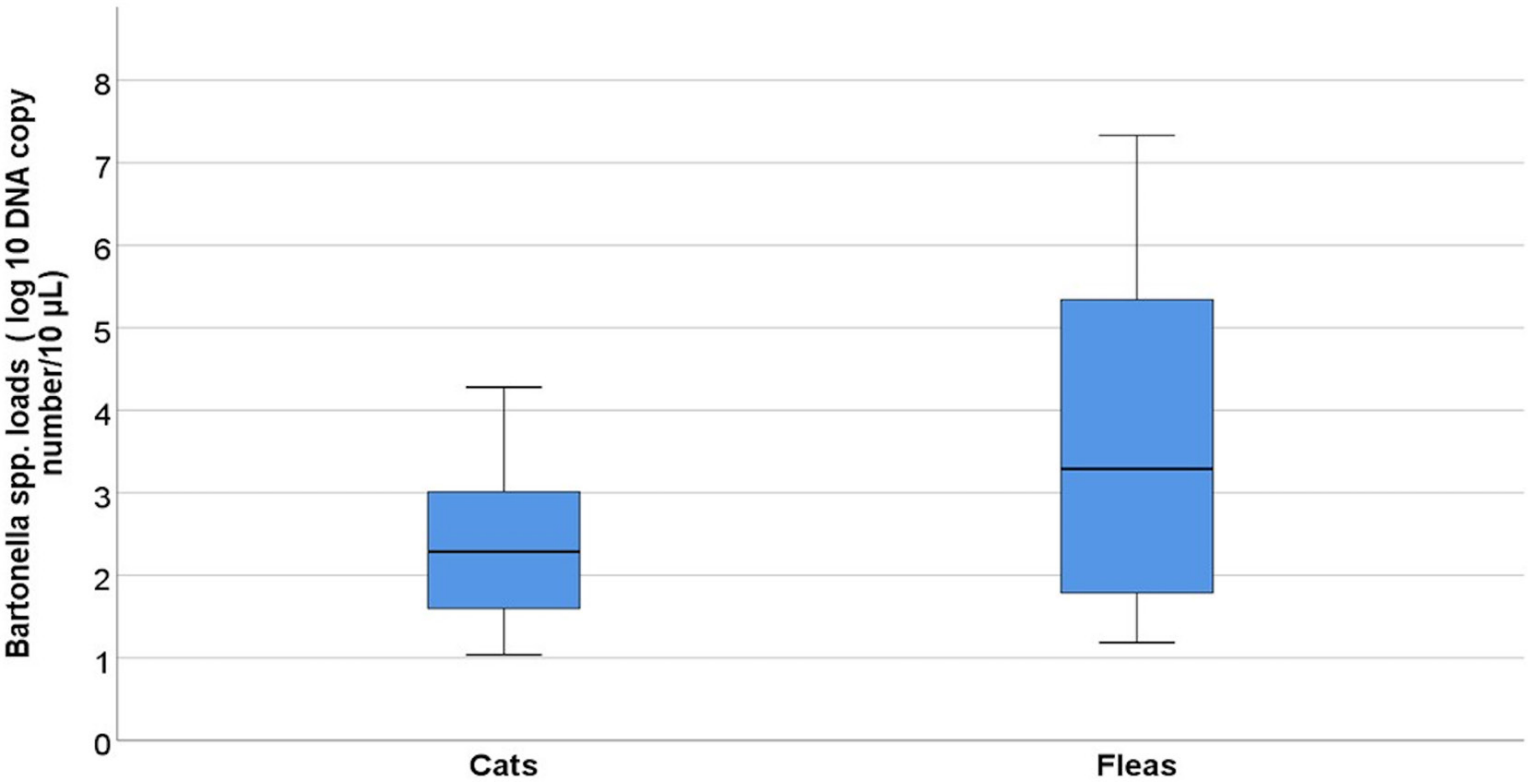
*Bartonella* spp. loads, expressed as _log10_ DNA copy number/10 μl, in blood and flea samples collected from cats from East (EA) and Southeast Asia (SEA). Boxes represent IQRs, and horizontal black thick lines represent median values. Vertical lines (whiskers) represent the distribution of maximum and minimum values (Mann–Whitney *U* test, *p =* .038)

**FIGURE 2:**
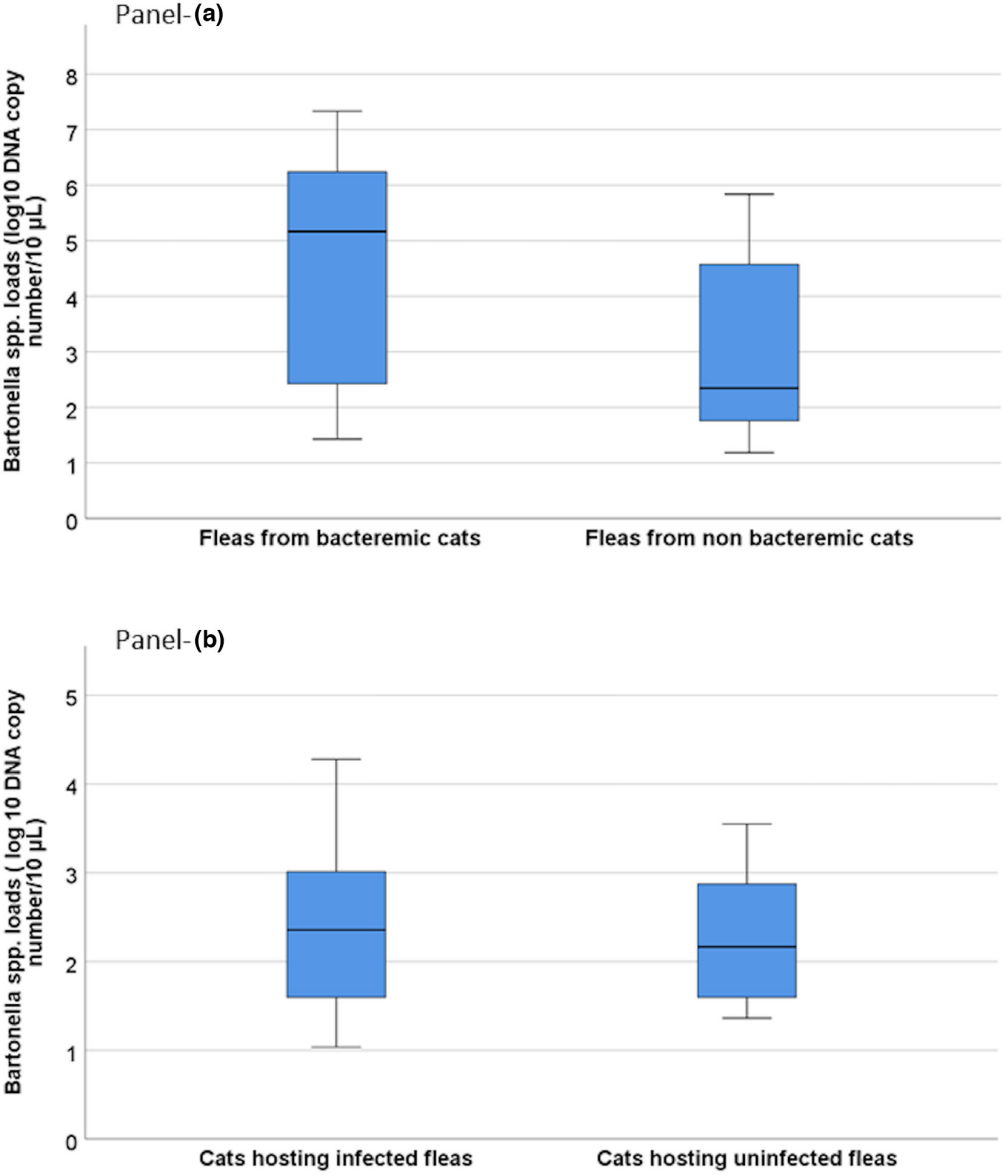
*Bartonella* spp. loads in cats and their fleas from East (EA) and Southeast Asia (SEA). Boxes represent IQRs, and horizontal black thick lines represent median values. Vertical lines (whiskers) represent the distribution of maximum and minimum values. The values on the *y* axis are expressed as _Log10_ DNA copy number/10 μl. Panel a represents the *Bartonella* spp. loads in fleas according to the infectious *status* of their cats (Mann–Whitney *U* test, *p =* .04*)*. Panel b represents the *Bartonella* spp. loads in cats according to infectious *status* of their fleas (Mann–Whitney *U* test, *p* = .97)

When comparing *Bartonella* spp. infectious rates, statistically significant higher *Bartonella* spp. DNA loads (*p* = .038) were detected in fleas than in host cats (Figure 1a,b). Noteworthy, fleas from bacteraemic cats had higher *Bartonella* spp. loads (range: 1.43–7.33 DNA copies/10 μl, mean: 4.5 ± SD 2.19; median: 5.15) than fleas from non‐bacteraemic ones (range: 1.18–5.84 DNA copies/10 μl, mean: 3.03 ± SD 1.6; median: 2.34; MWt, *p* < .05) (Figure 2a). No differences for *Bartonella* spp. loads were detected in host cats based on the infection status of their fleas (MWt, *p* = .97) (Figure 2b).

The *Bartonella* spp. DNA loads of cats that harboured infected fleas did not display significant differences from those of cats with *Bartonella*‐negative fleas (MWt, *p* = .748). Finally, low *Bartonella* spp. DNA loads were detected in the 3 positive fleas collected from dogs, with copy numbers ranging from 0.46 to 1.34 log_10_ (mean: 0.97 Log_10_ ± SD 0.46; median: 1.12) DNA copies/10 μl.

### 3.3 Detection and quantification of haemoplasma DNA

The efficiency of the two haemoplasma qPCR (16S rRNA) assays was more than 97% (Table 1). Haemoplasma DNA was detected in 16.13% (15/93, 95% CI = 8.65–23.60) of the cats, with *C*Mhm in 8 (8.6%, 95% CI = 2.90–14.30), *Mhf* in 6 (6.45%, 95% CI = 1.46–11.44) and mixed infection in one (1.08%) (Tables 3 and 4). In dogs, *Mhc* was the only species detected (4/96, 4.17%, 95% CI = 0.17–8.16). Furthermore, *C*Mhm was detected in all of the positive fleas from cats (7/93, 7.53%, 95% CI = 2.16–12.89) and *C*Mhp was detected in a flea from a dog (1.04%) (Table 3 and 4).

When comparing animal sources, haemoplasma occurrence was significantly more frequent in blood (*p* < .006, OR = 5.96) and flea (*p* = .02, OR = 7.73) samples from cats rather than from dogs (Table 5). No significant relationships were observed in cats for age (*p* = .17), gender (*p* = .70) or fever (*p* = .08) (Table 5).

**FIGURE 3:**
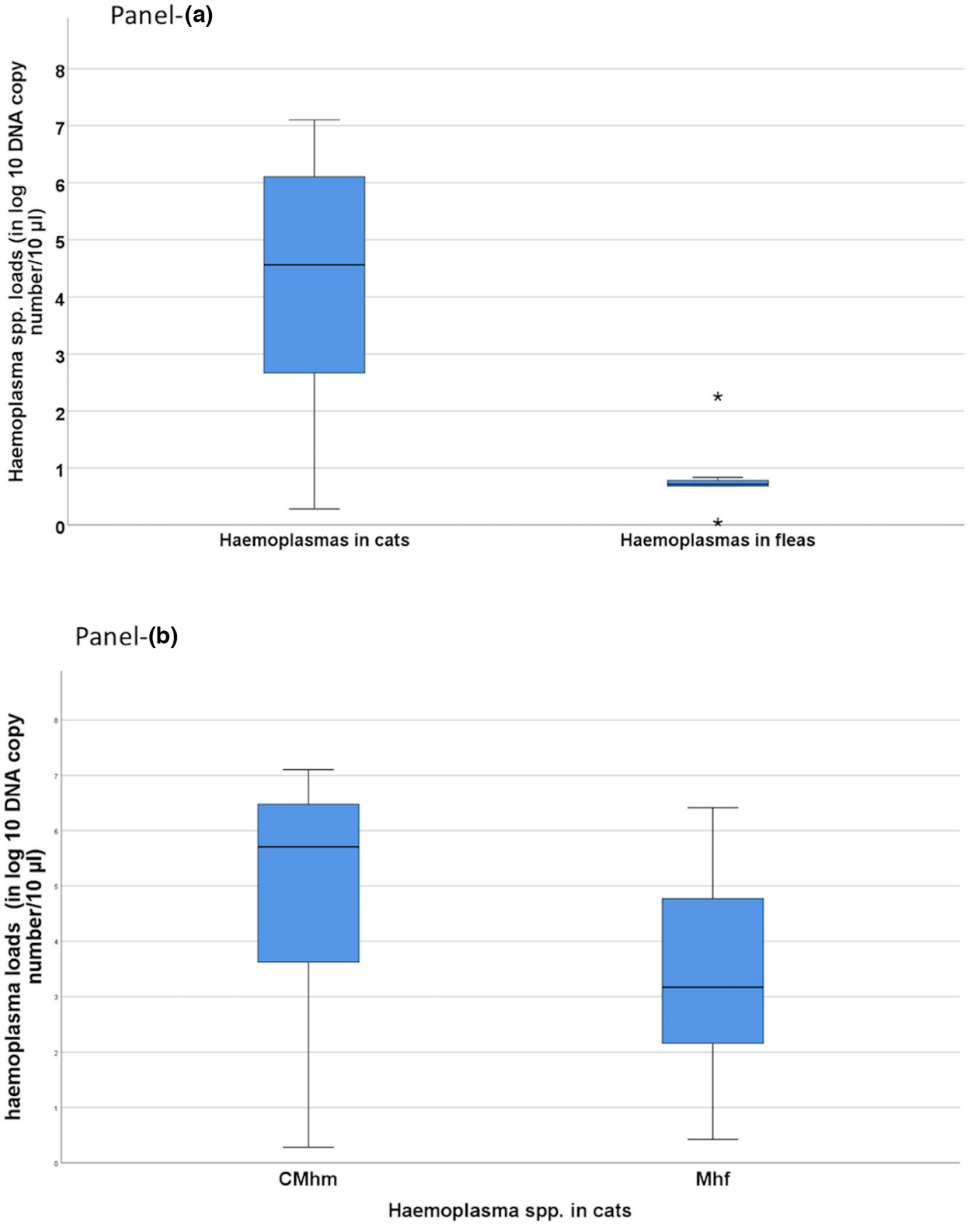
Haemoplasmas loads, expressed as _log10_ DNA copy number/10 μl, in samples from cats from East (EA) and Southeast Asia (SEA). Boxes represent IQRs, and horizontal black thick lines represent median values. Vertical lines (whiskers) represent the distribution of maximum and minimum values. Panel a, Haemoplasma loads in blood and fleas' samples from cats (Mann–Whitney *U* test, *p =* .003). Panel b, Positive cats grouped according to the detected haemoplasma species (Mann–Whitney *U* test, *p =* .21)

The haemoplasma DNA loads determined in the positive cats ranged from 0.283 to 7.10 log_10_ (mean: 4.20 log_10_ ± SD 2.28; median: 4.5) DNA copies/10 μl, with no significant differences for gender (MWt, *p* = .22), age (MWt, *p* = .51) or the presence of clinical signs (MWt, *p* > .1). The bacterial loads in haemoplasma‐infected fleas collected from cats ranged from 0.039 to 2.5 log_10_ (mean: 0.848 log_10_ ± SD 0.673; median: 0.716) DNA copies/10 μl with no difference for flea gender (MWt, *p* > .1). Statistically significant higher haemoplasma DNA loads (MWt, *p* = .003) were detected in host cats than in fleas (Figure 3a,b). Furthermore, no haemoplasma‐positive cats hosted infected fleas or vice versa. In dogs, haemoplasma DNA loads ranged from 0.57 to 7.14 Log_10_ DNA copies/10 μl (mean: 3.07 ± SD 2.43; median: 2.66) in dog hosts (all of them infected with *Mhc*), and 0.55 log_10_ DNA copies/10 μl in the sole positive flea (*Ct. felis*) from a dog that was infected with *C*Mhm.

## 4 DISCUSSION

In the present study, the occurrence and bacterial loads of *Bartonella* and haemotropic *Mycoplasma* species in both privately owned cats and dogs and their fleas from several EA and SEA countries were determined. *Ctenocephalides felis* was the dominant flea species infesting cats (97.85%) and dogs (75%) as already reported worldwide including Asia (Assarasakorn et al., 2012; Calvani et al., 2020; Colella et al., 2020; Nguyen et al., 2020; Rust, 2005; Tsai, Huang, et al., 2011; Wells et al., 2012). Moreover, *Ct. orientis*, also called the Asian flea, was detected in dogs (23.96%) as already described in Malaysia (Kernif et al., 2012) and Thailand (Changbunjong et al., 2009).

*Bartonella henselae* and possibly *B. clarridgeiae*, other than *B. koelerae*, are the agents of the cat scratch disease (CSD). Their prevalence rates might differ according to the geographic areas and climate conditions often overlapping the flea occurrence values (Yuan et al., 2011). In our study, the DNA of *Bartonella* species, including *B. henselae* and *B. clarridgeiae*, was detected in cats and fleas. The overall prevalence of *Bartonella* spp. in cat blood samples (21.51%) was consistent with previous surveys conducted in pet cats from Taiwan (19.1% to 22%) (Chang et al., 2006; Jensen et al., 2000; Maruyama et al., 2001) and Thailand (17%) (Assarasakorn et al., 2012;), lower than in the Philippines (28.9%) (Chomel et al., 1999) and Korea (33.3%) (Kim et al., 2009), and higher than in China (3.94% to 12.7%) (Yuan et al., 2011; Zhang et al., 2019). The prevalence of *Bartonella* spp. DNA (32.26%) in fleas from cats was similar to data available from Thailand, Japan and Australia (Assarasakorn et al., 2012; Barrs et al., 2010; Ishida et al., 2001). In detail, *B. henselae* genotype I was dominant in cats (13.98%) and their associated fleas (17.20%) while *B. henselae* genotype II was less frequent (1.08%) in both. A similar distribution of genotype I was reported from South East Asian countries (Chang et al., 2006; Chomel et al., 1999; Inoue et al., 2009; Jensen et al., 2000; Maruyama et al., 2000), in contrast to the European countries or USA where genotype II was dominant (Arvand et al., 2001; Chomel et al., 2002; Greco, Brianti, et al., 2019; Otranto et al., 2017). Furthermore, *B. clarridgeiae* occurrence in cats and their associated fleas (6.45%, 13.98%) overlapped that of previous studies (0.6 to 4.5%, 14%) in the area (Assarasakorn et al., 2012; Inoue et al., 2009; Kim et al., 2009).

Cats are the primary reservoir host for different *Bartonella* species that are mostly transmitted among cats by fleas (Breitschwerdt et al., 2010; Chomel et al., 1996; Chomel et al., 2006). Our study provides evidence that flea‐infested cats in urban areas of SEA represent a risk for *Bartonella* spp. infection for other cats and for their owners thus posing a potential threat to human health. When quantifying *Bartonella* spp. DNA, significant higher loads were detected in fleas rather than in host cats (*p* = .038), supporting the role of fleas as amplifier hosts. Indeed the capability of *B. henselae* to replicate in the gut of *Ct. felis* was previously observed (Bouhsira et al., 2013; Higgins et al., 1996; Rust & Dryden, 1997). Furthermore, significant higher DNA copy numbers were detected in *Bartonella*‐positive fleas collected from bacteraemic cats than from non‐bacteraemic ones (*p* < .05) possibly related to their role as *Bartonella* spp. accumulator following repeated blood meals on infected cat hosts (Bouhsira et al., 2013; Breitchwerdt & Kordick, 2000; Gutiérrez et al., 2015; Higgings et al., 1996; Rust & Dryden, 1997). The finding of *Bartonella* spp. negative cats hosting positive fleas suggests transient bacteraemia with undetectable levels of the pathogens at the time of arthropod sampling or early infection (Gutiérrez et al., 2015; La Scola et al., 2002; Lappin & Hawley, 2009). Nonetheless, *Bartonella*‐negative cats with positive fleas may also be possible flea transfer from infected to non‐infected cat.

Similar to a previous study performed in shelter cats from Brazil (Raimundo et al., 2019), age and gender were not risk factors for *Bartonella* infection in cats, although the sample selection method, which was based on the flea infestation status of the animals' enrolment, might have biased this observation. Indeed, it has been reported that the juvenile cats are more at risk to be found infected with CSD agents (Bergmans et al., 1996; Chomel et al., 1995; Greco, Brianti, et al., 2019; Zangwill et al., 1993).

Compared to cats, no risk for *Bartonella* spp. occurrence was observed in dogs being undetected in blood samples, and rare in their fleas. Similarly, in previous studies these bacteria were not detected in urban or rural dogs from Vietnam, Korea and China (Shenzhen) (Brenner et al., 2013; Suh et al., 2017; Zhang et al., 2019), and were found with low prevalence in Thailand (0.3%‐4.6) (Billeter et al., 2012; Inoue et al., 2009), Taiwan (1.7%) (Tsai, Chang, et al., 2011) and the Philippines (2.6%) (Singer et al., 2020). Conversely, higher occurrence (16%) was reported from a restricted sample of pet dogs that visited a veterinary teaching hospital in Korea as a likely result of selection bias (Kim et al., 2009). However, the occurrence of *Bartonella* spp. infection in the studied dogs cannot be excluded since *B. clarridgeiae* and *B. vinsonii*. subsp. *berkhoffii* infection in their associated fleas, *Ct. felis* and *Ct. orientis* was herein recorded similar to previous reports (Billeter et al., 2012; Kernif et al., 2012). To the best of our knowledge, *C*. B. merieuxii was herein detected for the first time in fleas, particularly, *Ct. felis*. This *Bartonella* species had already been detected in the blood of canids including dogs in Iran, jackals in Iraq and wolves in Italy (Chomel et al., 2012; Greco et al., 2021; Greco, Sazmand, et al., 2019).

The low frequency of *Bartonella* spp. infection generally recorded in domestic dogs suggests that these animals may be accidental hosts, rather than primary reservoirs in the cycle of *Bartonella* spp. (Breitschwerdt et al., 2010; Brenner et al., 2013; Chomel et al., 2006; Kaiser et al., 2011). Moreover, the higher frequencies (20% to 60%) recorded in stray and hunting dogs (Ebani et al., 2015; Greco, Sazmand, et al., 2019) compared to urban ones, as observed in the present study, suggest that wild wildlife and their ectoparasites are sources for the infection as documented by the detection of *Bartonella* spp. in wild canids, including foxes, wolves or jackals (Chomel et al., 2012; Greco et al., 2021; Hodžić et al., 2018).

Haemoplasma infection is common in cats and dogs worldwide (Biondo et al., 2009; Greco, Brianti, et al., 2019; Latrofa et al., 2020; Otranto et al., 2017; Sykes, 2010; Ravagnan et al., 2017; Roura et al., 2010). In the present study, 16.13% of the cats were positive for *Mycoplasma* spp. with *C*Mhm (8.6%) more frequent than *Mhf* (6.45%), similar to previous studies conducted in Thailand (Do et al., 2020, 2021; Kaewmongkol et al., 2020), South Korea (Hwang et al., 2016) and China (Kaewmongkol et al., 2017; Liu et al., 2016; Zhang et al., 2021). Moreover, CMhm was the only *Mycoplasma* species detected in fleas from cats (7.53%), in line with what was previously described in UK and Australia (Barrs et al., 2010). *Mycoplasma haemocanis* was the sole species identified in dogs (4.16%) similar to several studies conducted in Thailand (Kaewmongkol et al., 2017; Liu et al., 2016), Japan (Sasaki et al., 2008), Italy (Ravagnan et al., 2017) and the USA (Compton et al., 2012). However, *C*Mhm was detected only in one *Ct. felis* flea (1.16%) collected from a dog from the Philippines confirming the presence of the species in the area according to a previous report from the Thailand (Liu et al., 2016).

Altogether, our results show that cats from SEA are more at risk for haemoplasma infection (*p* < .01, OR = 5.96) rather than dogs. Worldwide, haemoplasma prevalence in cats varies according to several determinants including gender (male cats at higher risk of infection than female), lifestyle, infestation by ectoparasites or concurrent infections (i.e. FIV in cats) (Assarasakorn et al., 2012; Bergmann et al., 2017; Díaz‐Regañón et al., 2018; Do et al., 2020; Tasker et al., 2004; Willi et al., 2006). As far as risk factors, age (*p* = .1) and gender (*p* = .7) were not relevant for haemoplasma occurrence in cats as already observed in cats from Ontario (Kamrani et al., 2008).

Although it is still debatable how feline or canine hemoplasmas are transmitted, vector transmission through fleas or ticks (Lappin et al., 2006; Willi et al., 2007) has been hypothesized. In our study no haemoplasma‐positive cat had positive fleas or vice versa suggesting that the fleas may be feeding on different cats as already supposed (Assarasakorn et al., 2012). Furthermore, *C*Mhm DNA loads in fleas were substantially lower than (*p* = .003) those in cats, indicating a minor role of these ectoparasites as vectors, and a possible role of direct transmission (i.e. fighting) for these pathogens, as suggested in previous studies (Greco, Brianti, et al., 2019; Museux et al., 2009; Woods et al., 2005). Accordingly, *Mhc* prevalence was higher in Japanese fighting dogs than in other individuals of the same species, but with different lifestyles (Sasaki et al., 2008).

## 5 CONCLUSIONS

Privately owned cats and dogs living in East and Southeast Asia countries are exposed to *Bartonella* spp. and haemoplasma infections, with felines more likely to harbour these pathogens than canines (*p* < .0001). Noteworthy, the data presented strengthen that fleas serve as active vectors of *Bartonella* spp., but unlikely for haemoplasmas, in the area. Furthermore, we report the first detection of *C*.B. merieuxii in a female *C. felis* flea from an Indonesian dog. Also, this is the first study detecting different haemotropic *Mycoplasma* species from Indonesia (cats and their fleas), the Philippines (cat, dog, and fleas), Taiwan (cats and dogs) and Vietnam (cat fleas).

## ETHICS APPROVAL AND CONSENT TO PARTICIPATE

Approval for this study was obtained from the Animal Ethics Committee of the Veterinary Medicine Department of the University of Bari, Italy (Prot. no. 13/17). All animal owners have read, approved and signed owner informed consent containing information on study procedures and aims.

## Supporting information


Table S1

## Data Availability

The data that support the findings of this study are available from the corresponding author upon reasonable request.
