# Supplementary material for: Occurrence and bacterial loads of Bartonella and haemotropic Mycoplasma species in privately owned cats and dogs and their fleas from East and Southeast Asia
Source: Zoonoses Public Health. 2022 May 11;69(6):704–20. doi: 10.1111/zph.12959 (PMC9544368; doi:10.1111/zph.12959)
Supplement: Supplementary file 1 — Table S1 [file ZPH-69-704-s001.docx]

Table S: Distribution *of Bartonella* spp., haemoplasmas and co-infections according to the countries.

| Country /souce | *Bartonella* spp. | | | | | haemoplasmas | | | | | Co infection |
| --- | --- | --- | --- | --- | --- | --- | --- | --- | --- | --- | --- |
|  | Bh I | Bh II | Bc | Bvb | CBm | CMhm | *Mhf* | *Mhc* | *CMhp* | CMhm & *Mhf* |  |
| Philippines |  |  |  |  |  |  |  |  |  |  |  |
| C; Cat (29) |  |  | 3 |  |  | 4 | 4 |  |  | 1 | 1 (Bc & Mhf)  1 (Bc & *C*Mhm& *Mhf*) |
| D; Dog (45) |  |  |  |  |  |  |  | 1 |  |  |  |
| C; *C. felis* (20F, 9M) |  |  | 2F, 1M |  |  |  |  |  |  |  |  |
| D*; C. felis* (29F, 13M) |  |  |  |  |  |  |  |  | 1F |  |  |
| D; *C. orientis* (3F) |  |  |  |  |  |  |  |  |  |  |  |
| Malaysia |  |  |  |  |  |  |  |  |  |  |  |
| C; Cat (4) | 3 |  |  |  |  | 1 | 1 |  |  |  | 1 (Bh I & *Mhf)* |
| D; Dog (2) |  |  |  |  |  |  |  |  |  |  |  |
| C; *C. felis* (3F, 1M) |  |  |  |  |  |  |  |  |  |  |  |
| D; *C. felis* (2F) |  |  |  |  |  |  |  |  |  |  |  |
| Indonesia |  |  |  |  |  |  |  |  |  |  |  |
| C; Cat (20) | 8 |  | 2 |  |  | 2 |  |  |  |  |  |
| D; Dog (9) |  |  |  |  |  |  |  |  |  |  |  |
| C; *C. felis* (3M, 16F) | 9F, 1 M |  | 4 F |  |  | 2 (1F,1M) |  |  |  |  | 1F (Bc & CMhm) |
| C; *X. cheopis* (1M) | 1 M |  |  |  |  |  |  |  |  |  |  |
| D; *C. felis* (2F) |  |  |  |  | 1F |  |  |  |  |  |  |
| D; *C. orientis* (5F, 2M) |  |  |  |  |  |  |  |  |  |  |  |
| Taiwan |  |  |  |  |  |  |  |  |  |  |  |
| C; Cat (11) | 2 |  | 1 |  |  | 1 | 1 |  |  |  |  |
| D; Dog (9) |  |  |  |  |  |  |  | 2 |  |  |  |
| C; *C. felis* (7F, 4M) | 1F |  | 2F, 1M |  |  |  |  |  |  |  |  |
| D; *C. felis* (5F, 3M) |  |  |  |  |  |  |  |  |  |  |  |
| D; *C. orientis* (1F) |  |  |  |  |  |  |  |  |  |  |  |
| Thailand |  |  |  |  |  |  |  |  |  |  |  |
| C; Cat (5) |  |  |  |  |  |  |  |  |  |  |  |
| D; Dog (13) |  |  |  |  |  |  |  | 1 |  |  |  |
| C; *C. felis* (4F, 1M) | 1F |  | 1F |  |  |  |  |  |  |  |  |
| D; *C. felis* (5F, 4M) |  |  | 1F |  |  |  |  |  |  |  |  |
| D; *C. orientis* (1F, 3M |  |  |  | 1 M |  |  |  |  |  |  |  |
| Vietnam |  |  |  |  |  |  |  |  |  |  |  |
| C; Cat (16) |  |  |  |  |  |  |  |  |  |  |  |
| D; Dog (12) |  |  |  |  |  |  |  |  |  |  |  |
| C; *C. felis* (12F, 4M) | 3F |  |  |  |  | 5F |  |  |  |  | 2F (Bh I& *C*Mhm) |
| D; *C. canis* (1M) |  |  |  |  |  |  |  |  |  |  |  |
| D; *C. felis* (3F, 2M) |  |  |  |  |  |  |  |  |  |  |  |
| D; *C. orientis* (5F, 1M) |  |  |  |  |  |  |  |  |  |  |  |
| China |  |  |  |  |  |  |  |  |  |  |  |
| C; Cat (7) |  | 1 |  |  |  |  |  |  |  |  |  |
| D; Dog (6) |  |  |  |  |  |  |  |  |  |  |  |
| C; *C. felis* (2F, 5M) |  | 1F | 1F |  |  |  |  |  |  |  |  |
| D; *C. felis* (6F) |  |  |  |  |  |  |  |  |  |  |  |
| Singapore |  |  |  |  |  |  |  |  |  |  |  |
| C; Cat (1) |  |  |  |  |  |  |  |  |  |  |  |
| C; *C. felis* (1M) |  |  | 1M |  |  |  |  |  |  |  |  |

Legend: C: cat source, D: dog souce BhI, *B. henselae* genotype 1; BhII, *B. henselae* genotype 2; Bc: *B. clarridgeiae*; Bvb: *B. vinsonii* subsp. *berkhoffii*; *C*Bm: *Candidatus* Bartonella merieuxii *C*Mhm: C*andidatus* Mycoplasma haemominutum*; C*Mhp: C*andidatus* Mycoplasma haematoparvum; Mhc: *Mycoplasma canis*; Mhf: *Mycoplasma haemofelis*.
